# 381. Antibiotic Spectrum Index and Risk of *Clostridioides difficile* Infection

**DOI:** 10.1093/ofid/ofac492.459

**Published:** 2022-12-15

**Authors:** Michael J Ray, Kendall J Tucker, Jon P Furuno, Eric Lofgren, Luke Strnad, Jeffrey S Gerber, Jessina C McGregor

**Affiliations:** OSU/OHSU College of Pharmacy, Portland, Oregon; Wilkes University, Nesbitt School of Pharmacy, Old Forge, Pennsylvania; Oregon State University, Portland, Oregon; Washington State University, Pullman, WA; Oregon Health and Science University, Portland, Oregon; Children's Hospital of Philadelphia, Philadelphia, Pennsylvania; Oregon State University, Portland, Oregon

## Abstract

**Background:**

Antibiotic therapy is a known risk factor for *Clostridioides difficile* infection (CDI) and evidence suggests the magnitude of risk varies by spectrum of antibiotic activity and days of therapy (DOT). We quantified the risk of hospital-associated (HA) CDI associated with antibiotic therapy considering spectrum of activity and DOT.

**Methods:**

We performed a retrospective cohort study of adult (≥18 years) inpatient encounters at a 576-bed academic medical center in Portland, OR between January 2018 and February 2020. We excluded patients with hospital stays under 4 days and those known to have recurrent (CDI in the previous 12 weeks) or community-acquired (CDI diagnosis within first 3 days of hospitalization) CDI. The primary exposure of antibiotic utilization was measured using the Antibiotic Spectrum Index (ASI), where spectrum-based weights are applied to each agent and DOT over the time at risk, summed over the encounter, and divided by the number of antibiotic days. The primary outcome was HA-CDI. We estimated the independent association between aggregate ASI and HA-CDI using multivariable logistic regression.

**Results:**

Among 37,629 inpatient encounters, 68% patients received at least one antibiotic. We identified 159 cases of non-recurrent HA-CDI, which corresponds to 4.8 cases per 10,000 patient-days. Compared to those without HA-CDI, those with HA-CDI had a greater median number of distinct antibiotics prescribed (4 vs 1), greater median days of antibiotic therapy (22 vs 2), a greater median time at-risk (9 vs 6 days), and greater median Elixhauser Comorbidity Index (9 vs 4) (Kruskal-Wallis p-value < 0.0001 for all comparisons). There were no significant differences by age or sex. Patients with HA-CDI had a greater mean ASI per antibiotic day than those without (5.2 vs 3.2, Student’s t-test p < 0.0001). After adjusting for time at-risk and Elixhauser Comorbidity Index, each one-unit of ASI per antibiotic day was associated with 1.29 times increased odds of developing HA-CDI (Odds Ratio = 1.29, 95% Confidence Interval: 1.21 to 1.37).

**Conclusion:**

The Antibiotic Spectrum Index was strongly associated with HA-CDI. ASI may be a useful tool for quantifying the risk of CDI according to the specific antibiotic therapy received among hospitalized patients.

**Disclosures:**

**All Authors**: No reported disclosures.

